# Linking Multi-Dimensional Religiosity in Childhood and Later Adulthood: Implications for Later Life Health

**DOI:** 10.1177/01640275241267298

**Published:** 2024-08-01

**Authors:** Sara I. Hamm, Zachary Zimmer, Mary Beth Ofstedal

**Affiliations:** 1 Global Aging and Community Initiative, 3684 Mount Saint Vincent University, Halifax, NS, Canada; 2 Institute for Social Research, 1259 University of Michigan, Ann Arbor, MI, USA

**Keywords:** religion, spirituality, depression, disability, childhood, later adulthood

## Abstract

This study examines religiosity patterns across childhood and later adulthood and their associations with later-life health using an experimental module from the 2016 Health and Retirement Study (*N* = 1649; Mean Age = 64.0). Latent class analysis is used to categorize individuals by commonalities in religious attendance, religious identity, and spiritual identity. Cross-sectional and longitudinal associations are then explored using probable depression, disability, and mortality as health indicators. Results reveal complex patterns, often characterized by declining attendance and fluctuating identity. Relationships with health appear stronger in cross-sectional analyses, suggesting that some associations may be non-causal. Individuals with consistently strong religiosity show significantly better psychological health compared to their relatively non-religious counterparts. Moreover, the absence of religiosity in later adulthood is associated with an increased risk of mortality. Overall, the findings support the promotion of religiosity whilst acknowledging individual variations and highlighting the need for more individualistic approaches to the study of religion and health.

## Introduction

On-going population aging and increasing longevity has intensified the focus on behavioral and modifiable factors that can promote later-life health. Within this context, religiosity has emerged as a significant area of interest due to its associations with various health outcomes, including morbidity, psychological well-being, recovery from illness, and mortality (Koenig, 2013; Zimmer et al., 2016). Recognizing the role of religiosity in patient care, particularly for older individuals dealing with chronic illnesses or nearing the end of life, has led some to suggest the integration of religiosity into a comprehensive and patient-centered care strategy (Nelson-Becker, 2018). The empirical analyses performed by this paper aim to explore several evolving aspects of the extant literature on religiosity and later-life health. They identify common multi-dimensional patterns of religiosity across childhood and later adulthood and investigate their associations with later-life health outcomes.

## Literature Review

In the United States (US) and globally, older adults tend to participate in religious services more regularly and maintain stronger religious beliefs than younger adults (Zimmer et al., 2016). This age-related difference can be attributed to multiple factors. Some suggest that developmental processes may lead older adults to adopt a more religious outlook. Religion can provide a source of psychological comfort, offering hope and a sense of security when nearing the end of life (Hayward & Krause, 2016). It can also aid in coping with health declines, losses, and loneliness (Keonig et al., 2012), while providing social support and opportunities for maintaining social interactions (Zimmer et al., 2016). Cohort effects can also shape religious attitudes. The alternative spirituality movement of the 1970s emphasized personal expression over traditional religious practice. Consequently, the ‘Baby Boom’ generation has been noted for having less traditional views on religion and lower levels of religiosity in their later adulthood (Schwadel, 2011). Even so, others have argued for the influence of period effects, where changes in religiosity are tied to cultural and societal trends over time rather than to specific birth cohorts (Schwadel, 2011).

The importance of religiosity is underscored by a vast and long-standing body of research, dating back decades and even centuries, linking religiosity to health (Dearmer, 1909; Koenig et al., 2012). Studies have shown positive correlations between religiosity and numerous health measures, including cardiovascular disease, disability, kidney disease, chronic pain, cancer, and self-rated health (Koenig et al., 2012; Oman & Syme, 2018; Roger & Hatala, 2017). Religiosity has also been associated with mental and psychological well-being, reducing the number of anxiety and depressive symptoms (Garssen et al., 2020; Koenig, 2013; Roger & Hatala, 2017). Some scholars suggest that religiosity may moderate the relationship between physical and mental health by providing coping resources to help manage functional decline and chronic disease, leading to faster recovery times and reduced impact of various health conditions (Koenig et al., 2012; Oman & Syme, 2018). Demographic studies have also shown that regular religious service attendance is associated with a decreased risk of mortality; a relationship that persists even when accounting for several confounding factors (Hill et al., 2011; Idler et al., 2017).

Although no single theory has gained prominence in explaining the salutary link between religiosity and health, several mechanisms have been proposed. George et al. (2002) identified four main features of religiosity that are believed to promote health. First, religiosity can encourage healthier behaviors, such as abstaining from alcohol, maintaining a healthy diet, and promoting respect for the body. Second, religiosity can facilitate social support by providing greater opportunities to form friendships with like-minded individuals. Third, religiosity can cultivate various psychological resources, such as enhanced self-esteem and self-efficacy, which can help individuals to cope with stressful life events. Finally, religiosity can provide a sense of coherence and meaning to life, which can be beneficial to individuals seeking a sense of purpose or hope during stressful or challenging times (George et al., 2002).

While studies often report a positive connection between religiosity and health, contrasting findings also exist, highlighting the complexity of this relationship (e.g., Hayward & Krause, 2016; Maselko et al., 2009). These discrepancies arise from the multi-faceted nature of religiosity, which can include subjective, cognitive, behavioral, social, and cultural dimensions (Flannelly et al., 2004; Neff, 2006). Attendance at religious services is the most consistently used measure and has been associated with improved health outcomes, including reduced all-cause mortality and depressive symptoms (Idler et al., 2017; VanderWeele et al., 2022). These improvements are often attributed to the structured social support and sense of community inherent in religious services (VanderWeele et al., 2022). However, the complexity of religiosity and its measurement across different dimensions can lead to diverse effects on health.

A key distinction exists between public religiosity, expressed through formal institutional gatherings, and private religiosity, expressed through prayer and personal belief. Another important distinction is between religion and spirituality. Although these two concepts may overlap, religion typically involves organized practice and communal worship within an institution, whereas spirituality focuses on personal growth, meaning, and transcendence (Maselko et al., 2009). Both can provide individuals with a sense of purpose, meaning, and coping resources (Hayward & Krause, 2016). However, there is evidence to suggest that each may work through unique mechanisms. Maselko et al. (2009) distinguished between religious attendance and spirituality. They defined spirituality as being comprised of religious well-being, which refers to the quality of a person’s relationship with God or a higher power, and spiritual well-being, which refers to a person’s sense of meaning and purpose in life. Their findings suggest that religious attendance and spiritual well-being are associated with a reduced risk of depression, whereas religious well-being is associated with an increased risk. Specific spiritual practices, such as meditation, have also been explored for their health benefits. Meditation has been found to reduce stress and anxiety, maintain mental well-being, and positively affect biological outcomes like cell activity, heart rate, and blood pressure (Cahn & Polich, 2006; Ernest et al., 2008; Fang et al., 2010; Ledesma & Kumano, 2009). These findings suggest that religion and spirituality may exert alternative effects on health. However, neither concept has been very well-defined in the literature (Zimmer et al., 2016). In the following analysis, we aim to capture a range of subjective experiences with religiosity by measuring religious and spiritual identity, as well as attendance. Religious and spiritual identity are often used as indicators to understand how people identify with and experience religion and spirituality (Zagumny, 2013). By incorporating these two measures, we seek to capture the complex interactions between different facets of religiosity and their unique impacts on health.

Discrepant findings on the relationship between religiosity and health may also be explained by non-causal associations. For example, mobility restrictions associated with disability can impede religious service attendance (Maselko et al., 2012). Additionally, individuals experiencing depression may rely more heavily on religious coping mechanisms (Maselko et al., 2009). These examples highlight the risk of reverse causation when relying on cross-sectional data (Ferraro & Kelley-Moore, 2000; Maselko et al., 2012). Much of the existing literature has been cross-sectional in nature, limiting our ability to make causal inferences. While longitudinal studies cannot definitively establish causation, they do offer insights into potentially predictive relationships between religiosity and health (Flannelly et al., 2004).

Studies that examine religiosity across the life course suggest that maintaining consistent levels of religiosity from childhood to later adulthood is the most beneficial (Hwang et al., 2022). Compared to those with sporadic or minimal religious attendance throughout the life course, those who consistently attend religious services tend to experience fewer chronic conditions (Upenieks & Schafer, 2020), fewer depressive symptoms (Upenieks & Thomas, 2021), better cognitive health (Hill et al., 2020), and a reduced risk of mortality (Upenieks et al., 2021). Some have proposed an accumulative mechanism to explain these findings, suggesting that consistent religiosity across the life course can build a form of ‘religious capital’ that positively influences health (Iannaccone, 1990),

Typically, primary caregivers introduce religion to individuals during their childhoods, integrating them into a religious community (Tratner et al., 2020) This form of socialization lays the groundwork for religiosity throughout life (Upenieks & Schieman, 2022). However, while parents may provide the initial framework, religious beliefs can change or evolve as individuals navigate different life stages (Ingersoll-Dayton et al., 2002). A life course perspective views religiosity as dynamic, influenced by personal and societal experiences. Major life events like marriage, parenthood, career changes, and aging can either strengthen or diminish religious involvement (McNamara Berry et al., 2010). Ingersoll-Dayton et al. (2002) provided a way to theorize about these potential changes by identifying four distinct patterns among older American adults – stability, increasing, decreasing, and curvilinear – and noting that key life events like child rearing, adverse experiences, and disillusionment with church members can influence these trajectories. Their findings highlight the importance of recognizing the fluid and evolving nature of religiosity over time (Ingersoll-Dayton et al., 2002).

### The Present Study

The present study uses an experimental module from the 2016 Health and Retirement Study (HRS) to achieve two objectives. First, it aims to categorize distinct classes of religiosity among older American adults based on patterns of religious service attendance, religious identity, and spiritual identity in childhood and later adulthood. Second, it aims to examine the associations between the identified classes and later-life health using cross-sectional and two-year change data. While the first objective is somewhat exploratory, we expect to find several distinct patterns of religiosity, reflecting stability, increasing, and decreasing trajectories, as described by Ingersoll-Dayton et al. (2002). We also expect that individuals with consistently high religiosity across childhood and later adulthood will exhibit more favourable health outcomes.

## Research Design

### Data and Sample

The present study uses data from the Health and Retirement Study (HRS), a biennial survey following adults aged 50+ in the United States (US). The HRS began in 1992 with a cohort born between 1931 and 1941. Additional cohorts were added in 1998 to make it nationally representative of those aged 51+ living in the community. From the 2000 wave onwards, sample weights were added for nursing home residents, ensuring full representation of the older US population.

The core questionnaire covers many topics, including socio-demographics, economics, physical and psychological health, and family characteristics. Mortality data is obtained through interviews and linkages to the National Death Index. Each wave includes a distinct set of experimental modules, administered to a randomly selected sub-sample of respondents at the end of the core interview. The 2016 wave includes ten experimental modules, each administered to approximately one-tenth of the sample. The present study uses experimental module eight, which focuses on respondents’ religious life histories. Of the 20,912 respondents in the 2016 HRS wave, 1,767 were assigned and responded to module eight. After removing respondents with missing information on key variables, the final sample comprised 1,649 respondents. While the age distribution of module eight closely resembled that of the full HRS, the gender distribution contained a slightly higher proportion of males (48.5%) compared to the total sample (46.5%).

### Measures

To measure religiosity, we used six items capturing three dimensions: religious service attendance, religious identity, and spiritual identity. Each item was dichotomized into high and low levels to facilitate classification using Latent Class Analysis (LCA). Religious attendance was measured using questions: “*About how often did you attend religious services during your childhood?”* and “*About how often did you attend religious services in the past year?*” To maintain reasonable distributions, respondents who attended religious services at least once a week were coded as having ‘*regular*’ attendance and others were coded as having ‘*infrequent*’ attendance (Ofstedal et al., 2019; Upenieks et al., 2020). Religious identity was measured using questions asking: “*During your childhood, to what extent did you consider yourself to be a religious person?*” and “*To what extent do you consider yourself to be a religious person now?*” Respondents who considered themselves moderately or very religious were coded as having ‘*stronger*’ religious identity, and others were coded as having ‘*weaker*’ religious identity (Cotton et al., 2006; Neff, 2006). Spiritual identity was measured using questions that asked: “*During your childhood, to what extent did you consider yourself to be a spiritual person?*” and “*To what extent do you consider yourself to be a spiritual person now?*” Respondents who considered themselves to be moderately or very spiritual were coded as having ‘*stronger*’ spiritual identity, while others were coded as having ‘*weaker*’ spiritual identity (Cotton et al., 2006; Neff, 2006). Additional details on religiosity items and their respective distributions can be found in Supplemental Table 1.

Physical health was assessed in 2016 using the ‘Katz Index of Independence in Activities of Daily Living’, which evaluates six activities: walking, dressing, bathing, eating, getting in and out of bed, and using the toilet (Katz et al., 1963). Respondents who had difficulty on one or more of these activities were coded as having a disability (Katz et al., 1963). Psychological health was evaluated using eight items from the Center for Epidemiologic Studies – Depression Scale (CES-D; Radloff, 1977). Respondents were asked about their experiences over the past week with feeling depressed, perceiving activities as effortful, experiencing restless sleep, feeling happy, experiencing loneliness, finding joy in life, feeling sadness, and struggling to initiate activities. Two questions, specifically “*feeling happy*” and “*finding joy in life*” were reverse coded. Using a commonly used threshold (Karim et al., 2014), respondents reporting depressive symptomology on three or more items were categorized as having probable depression. To track changes over a two-year period, we replicated these measures in the 2018 wave while controlling for baseline disability and probable depression. Additionally, we considered self-assessed health at baseline, determined by the question, “*Would you say your health is excellent, very good, good, fair, or poor?*”, to account for other extraneous health issues (Jylhä, 2009). To address potential loss to follow-up due to factors related to baseline health and mortality, follow-up physical and psychological health measures included categories for ‘*deceased*’ and ‘*missing*’.

Our models also control for other potential confounders, including age, sex (0 = *male*, 1 = *female*), race (0 = *white*, 1 = *other*), college education (0 = *no college education*, 1 = *at least some college education*), partnership status (0 = *not partnered,* 1 = *partnered*), childhood health (0 = *good, very good, excellent*, 1 = *poor, fair*), and parental socio-economic status (SES; 0 = *about average, pretty well off financially*, 1 = *poor*).

### Analytical Strategy

We used LCA to identify and categorize a variable representing several common patterns of religiosity in childhood and later adulthood based on six religiosity indicators. LCA was chosen due to its ability to combine individuals into sub-groups based on their shared response patterns (McCutcheon, 1987), which was well-suited to address our first research objective. The optimal number of groups, or classes, is determined using an iterative expectation-maximization algorithm, maximizing likelihood parameters and evaluating model fit statistics such as the adjusted likelihood ratio test (G^2^), Akaike Information Criterion (AIC), sample-size adjusted Bayesian Information Criterion (BIC), and average posterior probability of class membership. The resulting output includes item response probabilities, representing the likelihood that class members score high on a specific indicator, and class probabilities, representing the likelihood of an individual belonging to a particular class (Nylund et al., 2007). Individuals are then assigned to the class they are most likely to belong based on their class probabilities. In this analysis, the average posterior probability for the best-fitting model was 0.90, indicating strong model fit (see Supplemental Table 2).

Each class is characterized by either having *‘regular’* or *‘infrequent’* religious attendance and *‘stronger’* or *‘weaker’* religious and spiritual identity. The determination of *‘regular/infrequent’* and *‘stronger/weaker’* classifications are based on item response probabilities of 0.75 or greater. For example, if individuals within a class have a 75% or greater probability of scoring high on religious identity, they are classified as having *‘stronger’* religious identity. Conversely, if individuals within a class have a 75% or greater probability of scoring low on religious identity, they are classified as having *‘weaker’* religious identity. If probabilities fall between these thresholds, the class is considered to have mixed item response. While cutoff values in LCA are somewhat arbitrary (Ulbricht et al., 2018), the thresholds applied in this study ensure that class membership closely aligns with the defining characteristics of each class and maintains class distinctiveness.

To assess cross-sectional associations between religiosity classes and health outcomes, we employ logistic regression. For longitudinal associations, we utilize multinomial logistic regression to accommodate the change in health status over a two-year period, which includes categories for *‘deceased’* and *‘missing,’* resulting in unordered categorical variables.

### Sensitivity Analyses

We performed a series of sensitivity analyses to ensure the robustness of our findings. These involved testing various LCA solutions, health outcomes, and categorizations of religiosity indicators. We adjusted the definitions of *‘stronger’* and *‘weaker’* identity, explored multi-level categorizations of religiosity indicators, utilized the full-scale range of our health outcome variables, and examined potential interaction effects between religiosity classes and baseline health. Additionally, we conducted analyses with and without missing data. None of these analyses changed the interpretation of results presented below.

## Results

### Sample Description

Table 1 provides descriptive statistics of the sample. The mean age was 64.0 years (Standard Deviation [SD] = 9.7; range: 50–100) with a higher proportion of females than males (51.6%). At baseline, 12.2% had probable depression and 13.4% were disabled. After accounting for mortality and missing data, 8.5% had probable depression and 11.1% were disabled at two-years follow-up. Over this period, 58 individuals died, representing 2.8% of the weighted baseline sample.

**Table 1.** Weighted Descriptive Statistics of Study Variables.

	Total sample
*N*	1649
Mean age (*SD*)	64.0 (9.7)
% Sex	
Male	48.4
Female	51.6
% Race	
White	80.3
Other	19.7
% College education	
No college education	39.3
Some college education	60.8
% Partnership status	
Not partnered	34.4
Partnered	65.7
% Childhood health	
Good, very good, excellent	94.8
Poor, fair	5.2
% Parental socioeconomic status	
Average or higher	77.3
Low	22.7
% Probable depression at baseline (3+ symptoms)	
Not depressed	87.8
Depressed	12.2
% Disability at baseline (1+ ADL limitation)	
Not disabled	86.6
Disabled	13.4
% Probable depression at follow-up (3+ symptoms)	
Not depressed	72.0
Depressed	8.5
Missing	16.6
Deceased	2.8
% Disability at follow-up (1+ ADL limitation)	
Not disabled	71.5
Disabled	11.1
Missing	14.7
Deceased	2.8

### Latent Class Analysis

Table 2 summarizes the LCA results, detailing seven distinct religiosity classes, ordered from most to least prevalent. Supplemental Table 2 presents model fit indices and Supplemental Table 3 displays item response probabilities. The first three classes consisted of individuals ranking consistently high or consistently low on most religiosity indicators. Class one (containing 23.3% of the study sample) ranked high on five out of six indicators, with the exception of religious attendance in later adulthood. Class two (20.2%) ranked high on all indicators, and class three (17.7%) ranked low on five out of six indicators, with the exception of religious attendance in childhood. The four remaining classes exhibited fluctuating patterns of change between childhood and later adulthood. Class four (12.1%) displayed a strengthening of religious and spiritual identity, while class five (11.4%) showed a decline in religious attendance and a weakening of religious identity. Classes six and seven maintained high levels of religiosity in one domain, where class six (10.5%) maintained stronger religious identities and class seven (4.9%) maintained stronger spiritual identities.

**Table 2.** Summarized Findings From Latent Class Analysis.

		Religiosity Class						
Number		1	2	3	4	5	6	7
Descriptor		High religiosity with infrequent attendance in later adulthood	High religiosity	Low religiosity	Religious and spiritual identity move from weaker to stronger	Attendance moves from regular to infrequent; religious identity moves from stronger to weaker	Stronger religious identity	Stronger spiritual identity
Dimension	Period							
Religious Attendance	Childhood	Regular	Regular	Mixed	Mixed	Regular	Regular	Mixed
	Later Adulthood	Infrequent	Regular	Infrequent	Mixed	Infrequent	Mixed	Infrequent
Religious Identity	Childhood	Stronger	Stronger	Weaker	Weaker	Stronger	Stronger	Weaker
	Later Adulthood	Stronger	Stronger	Weaker	Stronger	Weaker	Stronger	Mixed
Spiritual Identity	Childhood	Stronger	Stronger	Weaker	Weaker	Mixed	Weaker	Stronger
	Later Adulthood	Stronger	Stronger	Weaker	Stronger	Weaker	Mixed	Stronger
*N* (%)		384 (23.3)	333 (20.2)	291 (17.7)	200 (12.1)	188 (11.4)	173 (10.5)	81 (4.9)

*Note.* “Regular” and “Stronger” indicate probabilities of .75 or greater of being classified as high for that indicator. “Infrequent” and “Weaker” indicate probabilities of .75 or higher of being classified as low for that indicator. “Mixed” indicates neither “Regular” nor “Infrequent” or “Stronger” nor “Weaker” classification.

Across the seven religiosity classes, there are several overarching trends. None of the classes showed *‘infrequent’* attendance in childhood, with all being characterized as having either *‘regular’* attendance in childhood (classes one, two, five, and six) or mixed item response (classes three, four, and seven). For only one class (class two) was *‘regular’* service attendance maintained, indicating a predominant trend toward *‘infrequent’* attendance in later adulthood. However, this trend did not necessarily correspond with changes in religious or spiritual identity. Some classes reported *‘stronger’* religious identity in childhood that weakened in later adulthood (class five). Others reported *‘weaker’* spiritual identity in childhood that strengthened in later adulthood (class four). Some individuals maintained *‘stronger’* identities (classes one, two, six, and seven), whereas others maintained *‘weaker’* identities (class three).

Supplemental Table 4 provides detailed information on the distribution of the seven religiosity classes among select socio-demographic variables. *F*-tests compare the variation within each class to the variation within the overall sample for each variable. Compared to the overall sample, class one contained significantly more non-white individuals with at least some college education. Class two had a significantly higher proportion of females. Class three included significantly more individuals aged 50–64, with good or better childhood health, and average or higher parental SES. Class four had a significantly higher proportion of non-white individuals with low parental SES. Class five had significantly more white males without any college education. Class six included significantly more white individuals aged 65+. Finally, class seven comprised significantly more non-white females with poor or fair childhood health.

### Cross-Sectional Associations Between Religiosity Classes and Health

Table 3 presents strong cross-sectional associations between religiosity classes and physical and psychological health outcomes, controlling for other potential confounding factors. Those in class two, who ranked high on all religiosity indicators, served as our reference category and consistently showed the lowest odds of disability and probable depression compared to the other six religiosity classes. Those in class one, characterized by consistently high religiosity, except for religious attendance in later adulthood, and class five, who experienced declining religious attendance and identity, showed significantly higher odds of probable depression. Those in classes one and four, the latter of whom had strengthened their religious and spiritual identities, showed significantly higher odds of disability. Coefficients for classes three, four, and six, were also very robust and neared significance for probable depression. Individuals who were partnered, had poor or fair childhood health, or low parental SES showed significantly higher odds of probable depression. Conversely, individuals who were older, came from diverse racial backgrounds, had poor or fair childhood health, or low parental SES showed significantly higher odds of being disabled.

**Table 3.** Logistic Regression Predicting Probable Depression and Disability by Religiosity Class, with Odd Ratios (OR) and Confidence Intervals (CI).

	Probable Depression		Disability	
	OR	95% CI	OR	95% CI
Religiosity class (class 2 ref.)^a^				
Class 1	1.98*	[1.16, 3.37]	1.72*	[1.11, 2.98]
Class 3	1.91	[1.00, 3.66]	1.14	[0.65, 2.21]
Class 4	2.04	[1.00, 4.15]	2.09*	[1.22, 4.45]
Class 5	2.31*	[1.04, 5.15]	1.17	[0.60, 2.61]
Class 6	2.12	[0.92, 4.90]	1.90	[0.92, 4.22]
Class 7	2.08	[0.83, 5.22]	0.82	[0.39, 1.89]
Age	1.02	[0.99, 1.04]	1.04**	[1.02, 1.06]
Female	0.91	[0.58, 1.42]	1.17	[0.75, 1.79]
Not white	1.07	[0.67, 1.71]	1.90**	[1.23, 2.89]
Some college education	0.86	[0.56, 1.32]	1.11	[0.75, 1.79]
Partnered	1.26**	[1.12, 1.42]	1.10	[0.99, 1.23]
Poor/fair childhood health	3.76**	[1.98, 7.13]	3.69**	[2.04, 6.75]
Low parental SES	2.23**	[1.44, 3.46]	1.67*	[1.18, 2.75]
Constant	0.01	[0.00, 0.06]	0.00	[0.00, 0.02]
*N*	1600		1605	

*p < .05, **p < .01.

^a^Class 1 = High religiosity except for infrequent attendance in later adulthood; Class 2 = High religiosity; Class 3 = Low religiosity; Class 4 = Religious and spiritual identity move from weaker to stronger; Class 5 = Attendance moves from regular to infrequent; religious identity moves from stronger to weaker; Class 6 = Stronger religious identity; Class 7 = Stronger spiritual identity.

### Association Between Religiosity Classes and Two-Year Follow-Up Health

Table 4 displays the multinomial logistic regression results predicting the change in health status over a two-year period. The top panel lists predictions for probable depression, while the bottom panel lists predictions for disability. In both cases, the reference category is the absence of probable depression or disability and class two serves as the reference group. The longitudinal results differ considerably from our cross-sectional findings. While no significant associations were found between religiosity classes and disability, significant associations emerged for probable depression. Specifically, those in class three, characterized by consistently low religiosity except for childhood religious attendance, and class six, characterized by stronger religious but not spiritual identities, exhibited significantly higher odds of probable depression. However, none of the classes were significantly different from class two with regards to disability.

**Table 4.** Multinomial Logistic Regression Predicting Two-Year Follow-Up in Probable Depression and Disability by Religiosity Class, with Odd Ratios (OR) and Confidence Intervals (CI).

	OR	95% CI	OR	95% CI	OR	95% CI
	Probable Depression versus No Probable Depression		Deceased versus No Probable Depression		Missing versus No Probable Depression	
Religiosity classes (class 2 ref.)^a^						
Class 1	1.05	[0.51, 2.17]	2.42	[0.78, 7.54]	1.54	[0.92, 2.60]
Class 3	3.29**	[1.56, 6.94]	5.80*	[1.41, 23.88]	0.71	[0.37, 1.36]
Class 4	1.49	[0.58, 3.84]	2.85	[0.77, 10.49]	1.04	[0.55, 1.98]
Class 5	1.31	[0.52, 3.32]	4.94*	[1.46, 16.69]	1.15	[0.57, 2.34]
Class 6	2.42*	[1.04, 5.64]	2.15	[0.59, 7.85]	1.28	[0.69, 2.40]
Class 7	1.86	[0.60,5.78]	0.25	[0.03, 2.48]	1.02	[0.43, 2.38]
Age	1.00	[0.98, 1.02]	1.09***	[1.05, 1.13]	1.00	[0.98, 1.02]
Female	1.76*	[1.06, 2.92]	0.79	[0.34, 1.80]	0.78	[0.54, 1.13]
Not white	1.15	[0.60, 2.19]	0.93	[0.34, 2.54]	1.08	[0.69,1.69]
Some college education	1.01	[0.60, 1.70]	0.74	[0.31, 1.72]	0.70	[0.49, 1.01]
Partnered	1.07	[0.95, 1.21]	0.96	[0.79, 1.18]	1.08	[0.98, 1.19]
Poor/fair childhood health	0.99	[0.38, 2.55]	2.47	[0.69, 8.88]	1.54	[0.64, 3.73]
Low parental SES	1.74	[1.03, 2.91]	3.00**	[1.32, 6.81]	0.85	[0.55, 1.31]
Baseline probable depression	8.71***	[4.99, 15.21]	5.42***	[2.65, 11.10]	1.91*	[1.08, 3.38]
Poor/fair baseline self-assessed health	6.64***	[3.85, 11.45]	3.44**	[1.71, 6.95]	1.85**	[1.16, 2.92]
Constant	0.01	[0.00, 0.07]	0.00	[0.00, 0.00]	0.18	[0.05, 0.72]
*N*	1600					
	Disabled versus Not Disabled		Deceased versus Not Disabled		Missing versus Not Disabled	
Religiosity classes (class 2 ref.)^a^						
Class 1	1.03	[0.55, 1.90]	2.75	[0.91, 8.29]	1.91*	[1.07, 3.41]
Class 3	0.90	[0.43, 1.88]	5.10*	[1.16, 22.35]	0.76	[0.38, 1.55]
Class 4	0.70	[0.31, 1.60]	2.45	[0.68, 8.84]	0.98	[0.47, 2.03]
Class 5	0.97	[0.50, 1.91]	5.33**	[1.63, 17.39]	1.27	[0.61, 2.64]
Class 6	0.80	[0.34, 1.86]	1.85	[0.50, 6.83]	1.17	[0.58, 2.37]
Class 7	0.59	[0.20, 1.75]	0.26	[0.03, 2.51]	1.12	[0.45, 2.77]
Age	1.01	[0.99, 1.04	1.09**	[1.04, 1.13]	0.99	[0.97, 1.01]
Female	1.38	[0.84, 2.27]	0.71	[0.31, 1.60]	0.90	[0.60, 1.34]
Not white	1.51	[0.92, 2.48]	1.00	[0.37, 2.69]	1.22	[0.76, 1.96]
Some college education	0.79	[0.51, 1.21]	0.67	[0.28, 1.58]	0.66*	[0.45, 0.98]
Partnered	1.15*	[1.01, 1.30]	1.01	[0.81, 1.26]	1.12*	[1.00, 1.25]
Poor/fair childhood health	2.49*	[1.22, 5.07]	2.92	[0.72, 11.76]	1.77	[0.68, 4.63]
Low parental SES	1.11	[0.68, 1.81]	2.91*	[1.25, 6.77]	0.95	[0.60, 1.50]
Baseline disability	12.34**	[7.36, 20.66]	8.95**	[4.24, 18.91]	2.20*	[1.20, 4.03]
Poor/fair baseline self-assessed health	3.21**	[1.97, 5.24]	2.83**	[1.35, 5.92]	1.55	[0.95, 2.54]
Constant	0.01	[0.00, 0.07]	0.00	[0.00, 0.00]	0.25	[0.05, 1.21]
*N*	1605					

*p < .05, **p < .01, ***p < .001.

^a^Class 1 = High religiosity except for infrequent attendance in later adulthood; Class 2 = High religiosity; Class 3 = Low religiosity; Class 4 = Religious and spiritual identity move from weaker to stronger; Class 5 = Attendance moves from regular to infrequent; religious identity moves from stronger to weaker; Class 6 = Stronger religious identity; Class 7 = Stronger spiritual identity.

Other covariates, such as partnership status, childhood health, and baseline health, were significant predictors of changes in disability over a two-year period. Specifically, individuals who were partnered, had poor or fair childhood health, were disabled at baseline, or had poor or fair self-assessed health at baseline were significantly more likely to experience disability. Conversely, being female, having probable depression at baseline, or having poor or fair self-assessed health at baseline was significantly associated with probable depression. Significant associations were also observed between religiosity classes and mortality. Those in classes three and five, the latter of whom experienced a decline in their religious attendance and identity, showed significantly higher odds of mortality. Mortality was also predicted by age, parental SES, probable depression/disability at baseline, and poor or fair self-assessed health at baseline. With regards to missingness, individuals in class one were significantly more likely to be missing compared to class two. However, this association was only found in our model of disability. Probable depression, disability, and poor or fair self-assessed health at baseline were associated with follow-up missingness. Additionally, in our model of disability, individuals who were partnered were more likely to be missing, while those with at least some college education were less likely to be missing.

**Figure 1.**
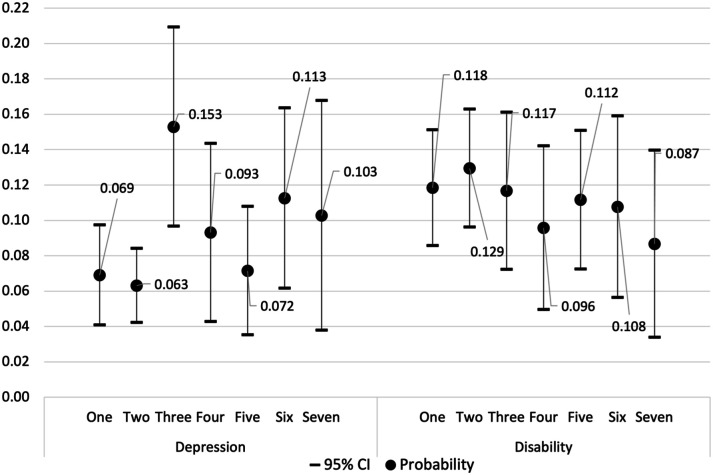
Predicted Probability of Follow-Up Probable Depression and Disability by Religiosity Class with 95% Confidence Intervals (CI). *Note.* Probabilities are calculated from the models shown in Table 4. Class 1 = High religiosity except for infrequent attendance in later adulthood; Class 2 = High religiosity; Class 3 = Low religiosity; Class 4 = Religious and spiritual identity move from weaker to stronger; Class 5 = Attendance moves from regular to infrequent; religious identity moves from stronger to weaker; Class 6 = Stronger religious identity; Class 7 = Stronger spiritual identity.

To aid our interpretation, predicted probabilities of follow-up disability and probable depression were calculated for each religiosity class, holding covariates constant at their mean values. Figure 1 illustrates the likelihood of a hypothetical ‘average’ individual experiencing follow-up disability or probable depression based on their class membership. It is important to note that the error bars within this figure do not necessarily align with the levels of significance shown in Table 4. This is because Table 4 shows the log odds of each outcome relative to those who are not depressed or disabled, while Figure 1 shows the probability of each outcome relative to any of the other three outcomes, including mortality and missingness. The results indicate that individuals in class three had a relatively higher probability of experiencing probable depression at the two-year follow-up (0.153) compared to the other six religiosity classes. This probability significantly differed from those in classes one and two (0.069 and 0.063, respectively). Interestingly, those in class five exhibited a relatively low probability of probable depression at the two-year follow-up (0.072), comparable to those in classes one and two. The probability of disability remained consistent across the seven religiosity classes, ranging only from 0.087 to 0.129.

## Discussion

Despite numerous reports highlighting the positive impacts of religiosity on later-life health, gaps exist within the extant literature. To address some of these gaps, we performed an empirical analysis using an experimental module from the 2016 Health and Retirement Study. We employed an LCA procedure to categorize sub-groups with common patterns of religiosity based on their religious service attendance, religious identity, and spiritual identity in childhood and later adulthood. Subsequently, we investigated their associations with later-life health.

We identified several distinct patterns of religiosity across childhood and later adulthood, which generally aligned with the findings of Ingersoll-Dayton et al. (2002). These patterns include stable, increasing, and decreasing religiosity over time. The most prevalent classes comprised individuals maintaining stable religiosity from childhood to later adulthood. Classes one and two displayed consistently high religiosity, while class three exhibited consistently low religiosity. Class one, the largest group, reflected findings from a previous study (Hwang et al., 2022) where many older adults were found to maintain strong religious and spiritual identities but stop attending religious services. Classes four and five comprised individuals whose religiosity had changed. Some moved away from their childhood religiosity (class five), while others saw an increase in religiosity (class four). Class seven maintained strong spiritual identities without strong religious identities, while class six retained strong religious identities without strong spiritual identities. Interestingly, class six showed mixed religious attendance in later adulthood, indicating strong religious beliefs without a robust institutional or spiritual investment (Maselko et al., 2009). This disconnection could render this group more susceptible to negative religious coping, particularly in the absence of strong ties to a larger religious community (Kidwai et al., 2014).

No class exhibited ‘*infrequent*’ religious attendance in childhood, while four reported having ‘*regular*’ attendance. The prevalence of ‘*regular*’ childhood attendance could reflect the strong societal emphasis placed on religion during respondents’ upbringing (Idler et al., 2017). However, ‘*regular*’ attendance did not necessarily translate into lifelong religious practice. Four classes exhibited ‘*infrequent*’ attendance in later adulthood, while only one maintained ‘*regular*’ attendance. This shift may be influenced by changing societal norms, evolving beliefs, or shifting priorities, influencing individuals’ religious attendance in later adulthood (Ingersoll-Dayton et al., 2002). Results also unveiled a small group who maintained strong spiritual but not religious identities in childhood and later adulthood (class seven). This finding aligned with the concept of ‘believing without belonging’ in previous literature, often attributed to the rise of individualism in modern society where personal beliefs are held independently of traditional religiosity (Koenig et al., 2012; Molteni & Biolcati, 2018). The identification of this class underscores the importance of distinguishing between religious and spiritual identity. Without this differentiation, class seven might have been inaccurately classified as having consistently low religiosity.

Our cross-sectional findings suggest that maintaining consistent religiosity from childhood to later adulthood is associated with better physical and psychological health. Those in class two, characterized by consistently high religiosity, showed significantly lower odds of probable depression compared to those in classes one (high religiosity except for infrequent attendance in later adulthood) and five (attendance moves from regular to infrequent; religious identity moves from stronger to weaker). This implies that maintaining consistently high religiosity may offer protection against depressive symptoms. However, the decline in religious attendance and identity observed in class five might also serve as a symptom of depression itself, indicating an inverse relationship (Maselko et al., 2012). Individuals in class two also displayed significantly lower odds of disability compared to those in classes one and four. However, the idea that a strengthening of religious and spiritual identity, as evidenced by class four, could contribute to disability is puzzling. One explanation is that increased religious and spiritual identity in later adulthood, coupled with declining religious service attendance, coincides with a pre-existing health condition or functional decline (Ferraro & Kelley-Moore, 2000; Maselko et al., 2012). In such cases, increasing religious and spiritual identity might function as a coping strategy for individuals to manage physical health challenges.

The direction of the relationship between religiosity and health is challenging to determine based on cross-sectional findings alone. To achieve a better understanding, we conducted analyses measuring the change in health status over a two-year period. The only consistent result was that individuals in class two (consistently high religiosity) had significantly lower odds of probable depression compared to those in the other six religiosity classes, most notably those in classes three and six. Those in class three, characterized by having consistently low religiosity, showed significantly higher odds of probable depression at two-years follow-up, supporting the notion that a complete absence of religiosity correlates with poorer mental health outcomes (Upenieks & Schieman, 2022). Interestingly, individuals in class six did not receive the same protective effects as those in class two. We suspect that the presence or absence of a strong spiritual identity could influence the relationship between religiosity and mental health outcomes. A strong spiritual identity typically involves feeling connected to a higher power and deriving a sense of meaning and purpose from one’s personal beliefs and practices (Maselko et al., 2009). Maintaining a strong spiritual identity, alongside a strong religious identity, could potentially mitigate the detrimental effects of negative religious coping and offer individuals a more balanced and non-punitive perspective when faced with life’s challenges.

Our multinomial results compare each of the seven religiosity classes to a single reference category (class two). Our predicted probabilities provide a different perspective, estimating the likelihood of experiencing probable depression or disability at two-years follow-up for each religiosity class directly, keeping covariates constant at their mean values. As such, transforming log-odds to probabilities leads to differences in results, particularly when comparing probabilities across categories. Our predicted probabilities indicated that those in classes one, two, and five had relatively low probabilities of experiencing probable depression at the two-year follow-up, while those in class three had a relatively high probability. Interestingly, the observation that class five had a lower likelihood of probable depression challenges the assumption that a decrease in religiosity necessarily leads to a higher rate of depressive symptoms. However, the high mortality rate (12.0%) among initially depressed individuals in class five could suggest survivor bias, potentially skewing results toward lower depression rates. Notably, those in classes one and two, characterized by relatively high and stable religiosity in both childhood and later adulthood, displayed the lowest probabilities of probable depression, whereas those in class three, characterized by consistently low religiosity, showed the highest probability.

Analyzing the change in health status over a two-year period revealed no significant differences in disability among the seven religiosity classes. Additionally, the predicted probabilities of experiencing disability at two-years follow-up was similar across all classes (Figure 1). These findings challenge previous literature suggesting that religiosity may serve as a protective factor against disability (Koenig, 2013). However, much of the existing literature has focused on religious attendance as the primary indicator of religiosity and/or has relied on cross-sectional data. Without longitudinal study designs and controls for baseline health, it remains uncertain whether religious individuals genuinely enjoy better functional health. While it is commonly assumed that religious involvement predicts health status, it is equally plausible that an individual’s health status may influence their level of religiosity (Maselko et al., 2012). Some longitudinal studies have included control measures, such as baseline disability and self-rated health, while others have not (Flannelly et al., 2004). Our study suggests that when examining the link between religiosity and disability longitudinally and accounting for baseline health, the previously observed associations weaken substantially.

There is some evidence to support the role of religiosity in relation to mortality. Despite only 2.8% of the weighted baseline sample dying between waves, our analyses revealed that those in class two had significantly lower odds of mortality compared to those in classes three and five. These results align with previous research indicating that older adults who have weaker religious connections in later adulthood – using the same religiosity dimensions as measured by this study – face an increased risk of mortality compared to those with stronger connections, regardless of their childhood religiosity (Hill et al., 2011). They also lend some support to the conclusions drawn by Upenieks and Thomas (2021), who argue that individuals raised in non-religious homes or who did not publicly practice religion in their childhood can still benefit from religiosity in later adulthood. We also observed that individuals who maintain consistently strong spiritual but not religious identities (class seven) have relatively lower odds of mortality. While this finding may not be reliable due to the small number of deaths that took place over time, it does suggest the potential salutary role of spirituality.

Overall, our findings suggest that promoting religiosity can be psychologically helpful to older adults with a strong inclination towards religion in their later adulthood. Healthcare professionals might consider engaging in discussions about and supporting patients’ religious life histories if it aligns with their personal preferences and beliefs. This might involve facilitating connections with larger religious communities or providing resources to foster greater spiritual growth. However, it is essential for clinicians to recognize the complexity and individualized nature of religiosity for their patients. Not everyone may benefit from maintaining or increasing their religiosity in later adulthood. For example, individuals who experienced a decline in religiosity (class five) exhibited comparable mental health outcomes to those who maintained consistently high religiosity over a two-year period (see Figure 1). As such, the findings highlight the importance of delivering personalized care and honoring patients’ choices, particularly with regards to their personal beliefs and practices. This aligns with a patient-centered care strategy when integrating religion and spirituality into healthcare (Nelson-Becker, 2018).

### Limitations

It is important to acknowledge the limitations of the present study. First, the reliance on a cross-sectional dataset means that religious life histories were captured at a single time point, potentially introducing memory bias. Questions about childhood religiosity may have been subject to inaccuracies due to the passage of time, current beliefs, or emotional states (Hill et al., 2020). As such, changes in religiosity between childhood and later adulthood may not have been fully captured. Second, the study only included health data at two time points, separated by a brief two-year gap. Incorporating additional health measures over a longer period would have offered a greater understanding of the long-term effects of religiosity on later-life health, as it is possible that changes in disability were unable to be detected within the studied timeframe. Third, some scholars have questioned the conditional independence assumption of LCA (Reboussin et al., 2008). In this study, childhood religiosity variables may have been locally dependent on current religiosity variables, potentially inflating model fit indices. Prospective accounts of childhood religiosity would have mitigated this limitation by measuring the two sets of variables more discretely. Finally, while the study provides valuable insights into the relationship between religiosity and health, its generalizability to younger generations of older Americans may be limited. Cultural and societal shifts over time raise questions about whether the associations between religiosity and health will persist in the future (Upenieks & Thomas, 2021). Continued research will be necessary to monitor the relationship among subsequent generations of older American adults.

### Conclusion

While this study is somewhat exploratory with respect to categorizing religiosity over time, it provides nuanced insights into the relationship between religiosity and later-life health. The findings suggest that maintaining consistently high and stable religiosity across childhood and later adulthood contributes to better psychological health in later adulthood, particularly when compared to those with low and stable religiosity. Nonetheless, there is little evidence that religiosity affects physical functioning. While there is some preliminary evidence for the positive impact of spirituality on psychological health and mortality, further research is still needed to validate this association and determine its underlying mechanisms. Our findings highlight the disparities between cross-sectional and longitudinal data, emphasizing the need for more longitudinal studies to better approximate causality. Altogether, this study highlights the importance of adopting a nuanced and individualistic approach towards the study of religion and health, and suggests that this relationship may not be adequately understood through simplistic or one-dimensional approaches (Pearce et al., 2013).

## Supplemental Material

sj-pdf-1-roa-10.1177_01640275241267298 – Supplemental Material for Linking Multi-Dimensional Religiosity in Childhood and Later Adulthood: Implications for Later Life Health Supplemental Material for Linking Multi-Dimensional Religiosity in Childhood and Later Adulthood: Implications for Later Life Health by Sara I. Hamm, Zachary Zimmer, and Mary Beth Ofstedal in Research on Aging
