# Supplementary material for: Linking Multi-Dimensional Religiosity in Childhood and Later Adulthood: Implications for Later Life Health
Source: Res Aging. 2024 Aug 1;47(2):91–102. doi: 10.1177/01640275241267298 (PMC11656630; doi:10.1177/01640275241267298)
Supplement: sj-pdf-1-roa-10.1177_01640275241267298 – Supplemental Material for Linking Multi-Dimensional Religiosity in Childhood and Later Adulthood: Implications for Later Life Health [file sj-pdf-1-roa-10.1177_01640275241267298.pdf]

Supplementary Materials

Supplementary Table 1. Weighted Distributions of Answers to Eight Items Used to Construct Religious Profiles in Childhood and Later Adulthood.

Item	Item wording	Item coding	% classified as 'regular' or 'stronger'
Childhood religious attendance	About how often did you attend religious services during your childhood?	More than once a week/Once a week = Regular (1)<br>Two or three times a month/One or more times a year/Not at all = Irregular (2)	73.3
Childhood religious identity	During your childhood, to what extent did you consider yourself a religious person?	Very religious/Moderately religious = High (1)<br>Slightly religious/Not religious at all =Low (2)	66.1
Childhood spiritual identity	During your childhood, to what extent did you consider yourself a spiritual person?	Very spiritual /Moderately spiritual = High (1)<br>Slightly spiritual/Not spiritual at all = Low (2)	54.0
Current religious attendance	About how often have you attended religious services during the past year?	More than once a week/Once a week = Regular (1)<br>Two or three times a month/One or more times a year/Not at all = Irregular (2)	33.4
Current religious identity	To what extent do you consider yourself a religious person now?	Very religious/Moderately religious = High (1)<br>Slightly religious/Not religious at all = Low (2)	64.3
Current spiritual identity	To what extent do you consider yourself a spiritual person now?	Very spiritual /Moderately spiritual = High (1)<br>Slightly spiritual/Not spiritual at all = Low (2)	68.8

Note. All questions are dichotomized into regular versus irregular/infrequent and weaker versus stronger categorizations. Responses are determined based on items from the 2016 Health and Retirement Study core questionnaire and its "religious life histories" module. N = 1,649.

Supplementary Table 2. Fit Statistics for Latent Class Analysis.

	# of Parameters	G^2	Adjusted BIC	AIC	Df	Change in G^2	Change in Df	Sig. Level ($p < .05$)	Avg. Predicted Probability
Independence	6	2259.14	2284.53	2271.14	57	-	-	-	1.00
Two Latent Classes	12	744.70	799.70	770.70	50	1514.44	7	14.07	0.94
Three Latent Classes	18	525.51	610.14	565.51	43	219.19	7	14.07	0.92
Four Latent Classes	24	211.36	325.59	265.36	36	314.15	7	14.07	0.89
Five Latent Classes	30	106.22	250.07	174.22	29	105.14	7	14.07	0.89
Six Latent Classes	36	60.48	233.95	142.48	22	45.74	7	14.07	0.88
**Seven Latent Classes**	**42**	**28.90**	**231.99**	**124.90**	**15**	**31.58**	**7**	**14.07**	**0.90**
Eight Latent Classes	48	23.18	255.89	133.18	8	5.72	7	14.07	0.83
Nine Latent Classes	54	8.69	271.02	132.69	1	14.49	7	14.07	0.82

Note. Best-fitting model is shown in **bold**.

Supplementary Table 3. Probability of Being Categorized as High in Each Religious Domain in Childhood and Later Adulthood.

Domain	Period	Class 1	Class 2	Class 3	Class 4	Class 5	Class 6	Class 7
Religious Attendance	Childhood	**0.788**	**0.932**	0.469	0.481	**0.869**	**0.887**	0.582
	Later Adulthood	**0.034**	**0.974**	**0.049**	0.571	**0.011**	0.385	**0.092**
Religious Identity	Childhood	**0.998**	**0.972**	**0.006**	**0.236**	**0.894**	**0.997**	**0.037**
	Later Adulthood	**0.874**	**0.994**	**0.047**	**0.955**	**0.054**	**0.995**	0.370
Spiritual Identity	Childhood	**0.988**	**0.944**	**0.003**	**0.044**	0.485	**0.068**	**0.978**
	Later Adulthood	**0.952**	**0.996**	**0.124**	**0.944**	**0.219**	0.563	**0.962**

Note. Item response probabilities of .75 or greater are classified as being high in each domain. Probabilities of .25 or lower are classified as low in each domain. Probabilities meeting either criteria are shown in **bold.**

Supplementary Table 4. Distribution of Class Across Socio-Demographic and Socio-Economic Characteristics.

Religiosity Class[a]	Class 1	Class 2	Class 3	Class 4	Class 5	Class 6	Class 7	Total
Age								
50-64	61.7	55.1	64.3	59.0	58.6	48.4	67.8	59.0
65+	38.4	44.9	35.7*	41.0	41.4	51.6**	32.3	41.0
Sex (%)								
Male	48.9	37.8	59.7	46.1	56.4	47.7	36.8	48.4
Female	51.1	62.3**	40.3	53.9	43.6*	52.3	63.2*	51.6
Race (%)								
White/Caucasian	76.7	80.3	82.7	74.8	87.5	88.2	69.0	80.3
Other	23.3*	19.7	17.3	25.2*	12.5**	11.8**	31.0**	19.7
College Education (%)								
No College Education	34.7	36.9	36.5	44.8	48.7	40.7	41.4	39.3
Some College Education	65.3*	63.1	63.5	55.2	51.3**	59.3	58.6	60.8
Partnership Status (%)								
Not Partnered	62.0	69.4	69.1	65.6	68.6	60.2	60.3	65.7
Partnered	38.1	30.6	30.9	34.4	31.4	39.8	39.7	34.4
Childhood Health (%)								
Good or Better	94.3	93.1	97.0	94.9	96.9	97.1	86.1	94.8
Poor/Fair	5.7	6.9	3.0*	5.2	3.1	2.9	13.9**	5.2
Parental SES (%)								
Average or Higher	78.9	75.4	84.3	65.2	80.1	80.1	69.4	77.3
Low	21.1	24.6	15.7**	34.9**	19.9	19.9	30.6	22.7

Note. F-test indicating whether proportions among specific variables differ across classes.
[a]Class 1 = high religiosity except for irregular/infrequent attendance in later adulthood; Class 2 = high religiosity on all indicators; Class 3 = low religiosity on most indicators except for mixed attendance in childhood; Class 4 = religious and spiritual identity change from weaker to stronger; Class 5 = attendance moves from regular to irregular/infrequent and religious identity from stronger to weaker; Class 6 = stronger religious identity in childhood and later adulthood; Class 7 = stronger spiritual identity in childhood and later adulthood.

* $p < 0.05$, ** $p < 0.01$
